# Relationship between BMI with percentage body fat and obesity in Singaporean adults – The Yishun Study

**DOI:** 10.1186/s12889-021-11070-7

**Published:** 2021-06-01

**Authors:** Kexun Kenneth Chen, Shiou-Liang Wee, Benedict Wei Jun Pang, Lay Khoon Lau, Khalid Abdul Jabbar, Wei Ting Seah, Tze Pin Ng

**Affiliations:** 1 Geriatric Education and Research Institute (GERI), 2 Yishun Central 2, Tower E Level 4 GERI Admin, Singapore, 768024 Singapore; 2 Faculty of Health and Social Sciences, Singapore Institute of Technology, Singapore, Singapore; 3 Programme of Health Services and System Research, Duke-National University of Singapore Graduate Medical School, Singapore, Singapore; 4 Department of Psychological Medicine, National University of Singapore, Singapore, Singapore

**Keywords:** Obesity, Percentage body fat, BMI, Prevalence

## Abstract

**Background:**

The main aim of this study was to the determine relationship between Body Mass Index (BMI) and percentage body fat (BF%) in Singaporean adults, derive a prediction model to estimate BF%, and to report population BF%. The secondary aim was to determine the prevalence of overweight and obesity based on BF% threshold and the new risk categories for obesity in Singaporean population.

**Methods:**

This was a population-based study of 542 community-dwelling Singaporeans (21–90 years old, 43.1% men). Anthropometry and body composition were assessed. Relationship between BMI and BF% were analysed using multiple regression models. Prevalence of overweight and obesity were estimated using WHO and Singapore Ministry of Health (MOH) Clinical Practice Guidelines for BMI classification, and BF% cut-off points of 25 and 35% for men and women respectively.

**Results:**

We derived a prediction model to estimate BF% based on BMI, age and sex. The current cohort of Singaporeans when compared to Caucasians in the US and Europe as well as a Singapore cohort from 20 years age have higher BF% when matched for BMI, age, and sex. The overall population-adjusted prevalence of obesity according to WHO International classification (BMI ≥30 kg/m^2^) was 12.9% (14.9% men; 11.0% women); and 26.6% (30.7% men; 22.8% women) according to the MOH classification (BMI ≥27.5 kg/m^2^). However, using the BF% cut-off (> 25% for men and > 35% for women) resulted in very high prevalence of obesity of 82.0% (80.2% men; 83.8% women).

**Conclusion:**

There is a large discrepancy between BF% and BMI measured obesity in Singaporean adults. The results confirmed that Singaporean adults have higher BF% at lower BMI compared to US and Europe white counterparts; and that BF% in our population has increased over two decades.

**Supplementary Information:**

The online version contains supplementary material available at 10.1186/s12889-021-11070-7.

## Introduction

Obesity is a complex and chronic condition [1], clinically defined as the accumulation of excess body fat to the extent that it may have adverse effects on health [2]. Obesity has long been associated with increased risks of mortality, cardiovascular diseases, diabetes, and cancer, and is associated with significant health and economic burden [3]. BMI has long been used to define obesity in adults. World Health Organization (WHO) recommends an international BMI cut-off point classification for adults: overweight is BMI 25–29·9 kg/m^2^ and obesity is BMI ≥30 kg/m^2^ [4]. In 2016, WHO reported the global prevalence of obesity at 11% in men and 15% in women [5]. Despite the relatively stable obesity prevalence in the US (30–34%) and UK (23–24%) between 2005 and 2015, the global prevalence has increased due to the rising trend in Asia (including China and India) which comprise a major portion of the world’s population [6]. Southeast Asia, with lower initial prevalence of obesity (2–15%), has also experienced increasing obesity over the last decade, in tandem with globalization, rapid urbanization, and increase in socio-economic status [7, 8].

Therefore, it is crucial to determine obesity or threshold of body fat that is associated with increased adverse health risk. Two commonly used methodology used to determine accumulation of body fat are waist circumference and BMI. Waist circumference (WC), measured at midpoint of the last palpable rib and top of iliac crest [9], has good correlation with abdominal adiposity, and strong association with cardiovascular mortality [10]. WC have different cut-off points between Europeans (102 cm for men and 88 cm for women) and Asians (90 cm for men and 80 cm for women) due to different body sizes [9]. BMI (body weight divided by height squared) is not a good indicator of body fat, as body weight comprises both fat and fat-free mass. Furthermore, the relationship between BMI, BF%, and body fat distribution differ with ethnicity [11, 12]. Asians are found to have a higher body fat percentage for the same age, gender, and BMI, when compared to European white population, and have higher prevalence of type 2 diabetes (T2DM) and increased cardiovascular risk at lower BMI values compared to European white population [13]. In a cross-sectional study of Malaysian women aged 40–59, prevalence of obesity was 72.8% based on BF% (BF% > 33) but only 20.6% when classified using BMI ≥ 30 kg/m^2^ [14]. Therefore, having a common BMI cut-off for obesity is not appropriate, as these cut-off points were derived from studies of the relationship between BMI, morbidity and mortality in the Western populations [4, 15].

In 2004, a WHO expert consultation was established to address the appropriate BMI classification for Asian populations [13]. After a series of analyses of BMI, body composition and risk factors of six Asian population data set, consensus was to retain the international BMI cut-off points for Asian populations due to the diverse ethnicity and wide range of cut-off point observed within Asian populations, and further recommended adding cut-off points of 23, 27·5, 32·5 and 37·5 kg/m^2^ as points for public health actions [13]. Under its current Ministry of Health (MOH) Clinical Practice Guidelines, Singapore adopted the cut-off point of 23 kg/m^2^ for overweight, and 27·5 kg/m^2^ for obesity [9], as Singaporeans have a higher prevalence of Type 2 diabetes and increased cardiovascular risk factors at BMI below 25 kg/m2 [16]. Therefore, measuring obesity based on an individual’s BF% may be a better indicator of health risks.

Various methods have been developed to measure BF%, including densitometry, dilution technique and dual energy X-ray absorptiometry (DXA). While WHO has a clear BMI cut-off for defining obesity, there is no clear consensus on the threshold for BF% for overweight and obesity. Previous studies have suggested that BF% greater than 25% for men and 35% for women is the threshold for diagnosing obesity, which were derived from corresponding BMI of 30 kg/m^2^ in Caucasians [17–19]. In a population study, Vietnamese women were reported to have lower BMI, body weight and fat mass than US White women [20]. However, the prevalence of BF% > 35 were similar between the US White women (54%) and Vietnamese women (53%) [20].

Singapore is a multiracial and multicultural country, consisting of 74.4% Chinese, 13.4% Malays, 9.0% Indians, and 3.2% of various other races [21]. In 2013, it was reported that among the three major ethnic group (i.e. Chinese, Malays, and Indians), Chinese had the lowest prevalence of obesity (BMI ≥ 30 kg/m^2^) at 5.9%, Indians at 14.0%, and Malays at 20.7% [22]. In a previous study, Singaporean Chinese was found to have higher cardiovascular risk at low levels of BMI [23]. Relationship between BMI and BF% in Singaporeans was found to be different from Caucasians, and also among the three major ethnic groups [24]. However, these studies were conducted about 20 years ago. In the recent WHO World Health Statistics, the Singapore population was reported to have similar mortality rate from cardiovascular diseases as Western populations [25]. With the increase in mean BMI in Asians [8], the relationships between BMI and BF% among Singaporeans have likely changed. As Singapore had adopted the use of BMI 23 kg/m^2^ and 27.5 kg/m^2^for overweight and obesity, the prevalence of obesity based on BMI 23 kg/m^2^ and 27.5 kg/m^2^should also be studied. The primary aim of this study was to the determine relationship between BMI and BF% in the multi-ethnic (Chinese, Malay and Indian) population of Singapore, derive a prediction model to estimate BF%, and to report population BF%. The secondary aim was to determine the prevalence of overweight and obesity based on BF% threshold and the new risk categories for obesity in our population.

## Methods

### Settings

Participants were recruited among community-dwelling adults (≥21 years) from a large north-eastern residential town of Yishun in Singapore, with residential population of 220,320 (49·4% men), with 12·2% older adults (≥65 years) [21]. This is similar to the overall Singapore residential population of 4·02 million (48·9% men), with 14·4% older adults (≥65 years) [21].

### Participants

Random sampling methodology was employed to obtain a representative sample of approximately 300 male and 300 female participants, filling quotas of 20–40 participants in each sex- and age-group (10-year age-groups between 21 and 60 years, 5-year age-groups after 60 years). Conventionally, the sample size of 30 or greater per age-group is sufficient for normative measures [26]. Between October 2017 and February 2019, using a two-stage random sampling method, 50% of all housing blocks were randomly selected, and a random 20% of the units in each block were approached for participant recruitment. Between March and November 2019, 50% of all housing blocks were randomly selected and all units were approached. Up to three eligible participants were recruited from each housing unit using a door-to-door recruitment method. Non-response units were re-contacted a second time at a different time of day on a later date. Older adults above 75 years old were additionally recruited through community sources and from a list of registered participants in four senior activity centres. Exclusion criteria were: individuals with disabilities, injuries, fractures or surgeries that affected function, neuromuscular, neurological and cognitive impairments, or more than five poorly controlled comorbidities. Pregnant women or those planning for pregnancy were also excluded. The estimated overall response rate was 39·0%. Ethics approval was obtained from the National Healthcare Group Domain Specific Review Board (2017/00212). All respondents gave informed consent before participation in the study.

### Measurements and data collection

Body weight to the nearest  0.1kg and height to the nearest 0.1 cm were measured using a digital balance and stadiometer (Seca, GmbH & Co. KG, Hamburg, Germany). Waist and hip circumferences were measured using a non-elastic, flexible measuring tape around the navel and widest part of the hips respectively. These measurements were conducted by trained researchers at the research center. All participants underwent a DXA scan of the whole body (Hologic Discovery Wi, Hologic, Marlborough, MA, USA). The DXA scan was conducted by experienced radiographers. Body composition information - lean mass, fat mass, and bone mineral content, were obtained from the scan.

### Overweight and obesity

Classification of overweight and obesity by BMI were derived using WHO international criteria [4], and Singapore MOH Obesity Clinical Practice Guidelines [9, 13]. Overweight and obesity were defined internationally as having a BMI 25·0–29·9 kg/m^2^, and BMI ≥30·0 kg/m^2^, respectively. Singapore MOH Clinical Practice Guidelines defined overweight as BMI 23·0–27·4 kg/m^2^ and obesity as BMI ≥27·5 kg/m^2^. The BF% cut-off points for obesity were set at 25% for men, and 35% for women [4, 17, 27]. Waist circumference (WC) for abdominal obesity was defined as above 80 cm for women, and above 90 cm for men in Singapore [9].

### Statistical analysis

All statistical analyses were performed using SPSS Statistics version 22·0 (IBM, Armonk, NY, USA). Relationship between BMI and BF% was analysed using forward-backwward stepwise linear regression models. BF% was considered the dependent variable; 1/BMI and age were independent variable. Data was analysed separately by sex. In exploratory analysis, the relationship between BMI and BF% was not linear, hence 1/BMI variable was used to linearise the data and to avoid the need for logarithmic conversion or the inclusion of power [28, 29]. Potential interaction variables were explored in model development and a forward-backward stepwise procedure was utilised for the development of the prediction equation models. Values are presented as mean ± standard deviation (SD), unless otherwise stated.

## Results

### Subjects

A total of 542 participants (43·1% men) aged 21 years and above were recruited for the study. Due to incomplete data from five participants, data from the remaining 537 participants (81·6% Chinese, 8·9% Malay, 6·7% Indians, and 2·8% from other races) were analysed. The ethnic distribution was similar to that of Singapore’s population [21]. Table 1 shows the demographic characteristics of the participants. As expected, men were taller and heavier, had lower BF%, higher fat-free mass, lower fat mass, and higher bone mineral content (*p* < 0·005). BMI was not significantly different between men and women (*p* = 0·071).

**Table 1:** Participant Demographic Characteristics

Variable	Men	Women	***P*** value
**Number of participants**	229	308	
**Age (yr)**	58·9 ± 19·1	58·4 ± 18·5	0·736
**Number of participants by age group**			
21–29	25	30	
30–39	26	30	
40–49	22	40	
50–59	18	41	
60–64	29	27	
65–69	22	36	
70–74	28	27	
75–79	32	34	
80+	27	43	
**Weight (kg)**	70·2 ± 15·4	58·8 ± 10·9	< 0·001
**Height (cm)**	166·6 ± 7·1	155·0 ± 6·4	< 0·001
**Waist Circumference (cm)**	91·4 ± 15·4	81·6 ± 10·8	< 0·001
**Overall BMI (kg/m**^**2**^**)**	25·2 ± 4·9	24·5 ± 4·2	0·071
**BMI by age-group**			
21–29	27·1 ± 8·2	22·5 ± 4·5	
30–39	28·0 ± 6·7	24·4 ± 4·7	
40–49	27·2 ± 3·8	25·7 ± 4·3	
50–59	25·7 ± 3·2	25·7 ± 5·5	
60–64	24·0 ± 2·9	24·4 ± 3·6	
65–69	24·1 ± 3·4	25·0 ± 3·0	
70–74	24·2 ± 3·2	22·9 ± 3·7	
75–79	23·7 ± 3·0	25·0 ± 3·5	
80+	23·4 ± 4·1	24·3 ± 4·0	
**Overall Body Fat Percentage (%)**	30·0 ± 5·7	39·7 ± 5·2	< 0·001
**Body Fat Percentage by age-group**			
21–29	29·6 ± 8·1	37·0 ± 6·4	
30–39	29·7 ± 6·7	37·7 ± 5·7	
40–49	31·0 ± 4·8	39·2 ± 4·7	
50–59	28·0 ± 4·3	40·0 ± 4·5	
60–64	29·1 ± 5·1	40·9 ± 4·9	
65–69	30·0 ± 5·0	41·0 ± 4·1	
70–74	30·9 ± 5·8	39·3 ± 5·0	
75–79	30·3 ± 4·9	41·2 ± 5·1	
80+	31·3 ± 5·4	40·6 ± 5·5	
**Fat mass (kg)**	21·0 ± 8·2	23·2 ± 6·6	< 0·005
**Fat mass Index (kg/m2)**	7·6 ± 2·9	9·7 ± 2·7	< 0·001
**Fat-free mass (kg)**	45·1 ± 8·9	32·6 ± 5·0	< 0·001
**Fat-free mass index (kg/m2)**	16·2 ± 2·3	13·6 ± 1·8	< 0·001
**Bone mineral content (kg)**	2·40 ± 0·43	1·88 ± 0·36	< 0·001

Values are mean ± standard deviation, or actual number of participants

### Relationship between BMI and BF%

**Fig. 1:**
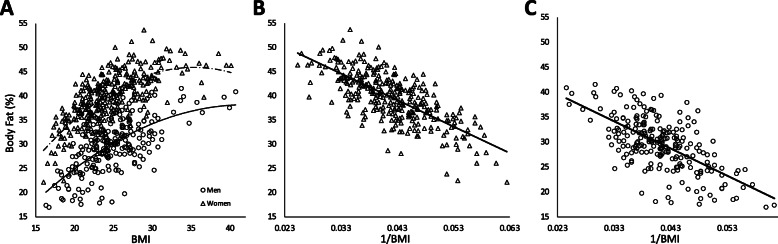
**a** Curvilinear relationship between BF% versus BMI **b** Linearize relationship between BF% and 1/BMI in women; *y = − 547.66 (x) + 62.696; R*^*2*^ = 0.52 **c** Linearize relationship between BF% and 1/BMI in men; *y = − 545.14 (x) + 52.181; R*^*2*^ *= 0.42;* ○: men; ∆: women

For the BF% prediction equation model, only data from the Chinese ethnic group (*n* = 438) was analyzed, as the ample sizes for Malay, Indian, and other races were too small. The relationship between BMI and BF% was curvilinear (Fig. 1a). We replaced BMI with 1/BMI as the independent variable to linearize the relationship (Fig. 1b & c). The regression models with 1/BMI provided higher multiple *R* and *SEE* values, compared to logarithmic transformed BMI values, as was reported previously [30]. Table 2 shows the regression coefficients of the stepwise multiple regression. The final prediction equation derived was,

$$ \mathrm{Men}:\mathrm{Percentage}\ \mathrm{Body}\ \mathrm{Fat}=49.818+0\cdotp 089\ \left(\mathrm{Age}\right)-619\cdotp 808\ \left(1/\mathrm{BMI}\right) $$

$$ \mathrm{Women}:\mathrm{Percentage}\ \mathrm{Body}\ \mathrm{Fat}=58.159+0\cdotp 051\ \left(\mathrm{Age}\right)-516\cdotp 401\ \left(1/\mathrm{BMI}\right) $$

**Table 2:** Regression coefficient of the stepwise multiple regression of body fat percentage as dependent variable

	1/BMI		Age		Intercept		***SEE***
	β	SE	β	SE	β	SE	
**Men**	− 575·291	47.401	–	–	53.430	2.010	3.86
	−619.808	43.923	0·089	0·015	49·818	1·929	3·53
**Women**	− 538.513	32.062	–	–	62·167	1.429	3.52
	−516.401	32.062	0.051	0·012	58.159	1.651	3·39

*1/BMI* 1 divided by Body Mass Index, *SE* Standard Error, *SEE* Standard Error of Estimate

where multiple *R* = 0.73, SEE = 3.69% body fat for men (*p* < 0.05), and multiple *R* = 0.75 and SEE = 3.45% body fat for women (*p* < 0.05). Based on the estimated parameters of these equations, BF% values corresponding with BMI for men and women were derived (Table 3). Estimated BF% of White, Japanese, and Vietnamese were derived from studies that published ethnicity specific equation models [30, 31]. Comparing estimated BF% from this study and from the 1998 National Heath Survey using equations published [24], men and women in 1998 were found to have lower BF% across all ages, ethnicity, and BMI categories, except for BMI 35·0 kg/m^2^ and above (Supplementary Table**)**.

**Table 3:** Estimated body fat percentage based on BMI of Singapore Chinese compared with other ethnicities

	Men					Women				
	BMI of 18·5	BMI of 25	BMI of 30	BMI of 35	BMI of 40	BMI of 18·5	BMI of 25	BMI of 30	BMI of 35	BMI of 40
**20-39y**										
Chinese	19·0	27·7	31·8	34·8	37.0	31·8	39·0	42.5	44·9	46.8
White [29]	14·5	23·9	29·8	33·3	35·9	26·9	37·0	41·8	45·2	47·7
Japanese [29]	12·8	23·2	28·1	31·6	34·3	24·6	35·2	40·2	43·8	46·5
Vietnamese [30]	18·3	26·5	29·8	30·6	28·8	29·2	37·4	40·7	41·5	39·7
**40-59y**										
Chinese	20.8	29/5	33·6	36·6	38·8	32.8	40.1	43·5	46·0	47·8
White [29]	15·6	25·4	30·0	33·3	35·8	27·5	37·4	42·2	45·6	48·1
Japanese [29]	13·4	23·8	28·7	32·2	34·9	25	35·5	40·2	44·1	46·8
Vietnamese [30]	19·1	27·2	30·5	31·5	29·7	30·1	38·5	41·8	42·4	40·6
**60-79y**										
Chinese	22·7	31·3	35·3	38·2	40·4	33·8	41·1	44·5	47·0	48·8
White [29]	19·0	28·0	32·3	35·3	37·6	31·0	39·9	44·1	47·1	49·4
Japanese [29]	13·9	24·3	29·3	32·8	35·4	25·3	35·8	40·9	44·4	47·1
Vietnamese [30]	20·1	28·2	31·6	32·4	30·6	31·0	39·1	42·5	43·3	41·5

Estimated body fat percentage calculated centering on the ages of 30, 50, and 70 years

### Prevalence of overweight and obesity

The prevalence of overweight and obesity are presented in Table 4. According to WHO International BMI classification, the overall population-adjusted prevalence of overweight was 34·4% (39·1% men; 29·9% women), and obesity was 12·9% (14·9% men; 11·0% women). Using the MOH classification, the prevalence of overweight was 41·8% (44·5% men; 39·3% women) and obesity 26·6% (30·7% men; 22·8% women). Using WHO proposed BF% cut-off, prevalence of obesity increased to 82·0% overall (80·2% men; 83·8% women). Using WC criteria, prevalence of abdominal obesity was 59·1% (55·7% men, 62·3% women).

**Table 4:** Sample and population-age adjusted prevalence of overweight and obesity based on BMI, BF and WC

	Sample Estimates					Population-Adjusted Estimates				
	Overall	21-59 yrs	≥60 yrs	≥65 yrs	≥75 yrs	Overall	21-59 yrs	≥60 yrs	≥65 yrs	≥75 yrs
**Total**										
Overweight	33·9	32·3	34·9	32·7	30·1	34·4	33·4	37·0	33·4	30·0
Obese	9·5	15·9	4·6	4·8	6·6	12·9	16·2	3·7	3·7	6·5
BF%	83·2	81·0	84·4	85·3	83·8	82·0	80·9	85·2	87·5	84·0
WC	63·9	55·2	70·0	70·9	70·6	59·1	55·6	68·8	69·9	70·2
OW_MOH_	42·8	39·2	45·3	44·2	45·6	41·8	40·8	46·0	44·2	45·4
OB_MOH_	21·8	29·3	16·0	15·5	14·0	26·6	30·3	16·3	15·4	14·0
**Men**										
Overweight	37·6	39·6	36·2	32·1	29·3	39·1	39·3	38·6	31·8	28·5
Obese	9·6	19·8	2·9	3·7	3·4	14·9	19·4	2·0	3·0	3·4
BF%	81·2	79·1	82·6	85·3	81·0	80·2	79·1	83·2	88·9	80·2
WC	54·1	54·9	53·6	54·1	50·0	55·7	56·7	52·9	53·6	49·1
OW_MOH_	45·4	41·8	47·8	45·0	43·1	44·5	42·6	50·0	45·5	41·8
OB_MOH_	21·8	37·4	11·6	11·0	8·6	30·7	37·4	11·6	10·4	8·7
**Women**										
Overweight	31·2	27·7	34·1	33·6	31·2	29·9	27·8	35·5	34·9	31·2
Obese	9·4	13·5	6·0	5·7	9·1	11·0	13·1	5·2	4·2	8·9
BF%	84·7	82·3	86·8	86·4	87·0	83·8	82·6	87·1	86·2	86·9
WC	71·1	55·3	84·4	85·0	87·0	62·3	54·6	83·4	84·3	86·9
OW_MOH_	40·9	40·9	37·6	43·7	44·3	39·3	38·2	42·3	43·0	48·2
OB_MOH_	21·8	24·1	19·8	19·3	18·2	22·8	23·6	20·6	19·8	18·1

Overweight (BMI 25·0–29·9 kg/m^2^) and obesity (BMI ≥30·0 kg/m^2^) classification based on WHO international classification.; BF% (body fat percentage) - Men: 25%; Women: 30%; WC (waist circumference) - Men: 90 cm; Women: 80 cm; OW_MOH_ (BMI 23·0–27·4 kg/m2) and OB_MOH_ (BMI ≥27·5 kg/m^2^) classification uses the Singapore MOH Clinical Practice Guidelines BMI classification

## Discussion

### Percentage body fat

In this study, we established the relationship between BMI and BF% in Singapore Chinese adults. We compared the estimated BF% with other ethnicity, and also with an earlier study on Singapore population from 20 years ago. Comparing among ethnicities, Singapore Chinese were found to have higher BF% compared to Caucasian. This supports the findings from the Singapore study in 2000 [24] and other reports that some Asians population have greater fat mass than Caucasians [14, 32–34]. However, there are other contrasting findings from other Asian populations, such as Vietnamese [31] and Polynesian [35] population that for similar sex, age, and BMI, the BF% was lower compared to Caucasians – showing the ethnic diversity in percentage body fat in Asia.

Our finding updates the 2000 report [24] in that current cohort of Singaporeans have higher BF% at matching BMI, age and sex compared to the cohort from 20 years ago [24]. The changes among Singaporeans may be due to changes in energy balance. Average daily energy intake increased 10·3% from 2004 to 2010 with majority (59·4%) of the population exceeding the daily recommended energy intake [36]. This increase in energy intake was not offset by the subsequent 5% reduction in average daily energy intake between 2010 to 2018 [37].

### Prevalence of overweight and obesity

The population-adjusted prevalence of overweight and obesity of Singaporeans varied according to the classification used. Prevalence of overweight was 34.4 and 41.8%, obesity was at 12.9 and 26.6%, when using WHO international BMI classification and MOH classification respectively. When adopting BF% criteria, population-adjusted obesity prevalence was substantially higher at 82.0% (Table 4). The corresponding high population-adjusted prevalence of WC > 80 cm for women and > 90 cm for men (59.1% overall, 55.7% for men and 62.3% for women in Table 4) suggests that central obesity account for much of this excess body fat in our population. Such substantial higher prevalence with BF% criteria had also been reported in the Vietnamese [31], and Saudi adults [38]. BMI was found to underestimate prevalence of obesity by about 50% when compared to BF% [38, 39]. This is in agreement with our results, where prevalence of overweight and obesity was found to be 43.4% based on BMI and prevalence was 83.2% based on BF% cutoff point. It is well-known that BMI, though highly specific, has low to moderate sensitivity when defining obesity and underestimate prevalence of adult excess body fat, particularly in Asians [31, 40]. While the Chinese and Koreans have proposed population-specific BF% cut-off [33, 41], there is yet no Asian consensus in BF% cut-off point. Our finding is a step towards such a consensus.

A previous study reported that the lowest all-cause mortality rate in Singapore Chinese was at BMI 18·5–19·9 kg/m^2^, with mortality rate significantly increased at BMI ≥26·0 kg/m^2^ for non-smokers [16], which is lower than the WHO Asian recommendation [13]. Using our derived equation, BMI 18·5–19·9 kg/m^2^ equates to BF% of 20·9–23·2% in Chinese men and 32·8–34·8% in Chinese women aged 50 years. At BMI 26 kg/m^2^, BF% equates to 30·4% in Chinese men and 40·9% in Chinese women, which is about 5% higher than the WHO BF% cut-off. Using the criteria of BF% ≥30·4% in men and ≥ 40·9% in women, 45% of men and 44% of women have increased mortality risk. These estimates are much higher than the prevalence based on BMI ≥30 kg/m^2^, but much lower than using the WHO BF% cutoff. Differences in prevalence based on BMI is expected because the relationship between BMI and body fat content varies according to body build and proportion [2]. People with low relative sitting height (i.e. length from the superior midline of the head to the sitting surface) will have a relatively low BMI compared to their BF% [42], but our Asian population have high relative sitting height [43]. The smaller body frame of Singapore Chinese partially contributed to their having higher BF% at the same BMI [43]. Physical inactivity is likely another contributing factor. The 2010 National Health Survey found 39·1% of Singaporeans did not meet the recommended physical activity guidelines [44]. The increase in energy intake and lack of physical activity could explain the high BF% in Singaporeans. Such high BF% in Singaporeans may explain the leading contributions to disease burden by cardiovascular disease and cancer [45]. Our study suggests that WHO international and local Health Ministry BMI classification still underestimated the obesity prevalence in Singapore [24]. Given the high discrepancy between prevalence of obesity using BMI versus BF%, the prediction equations for BF% from BMI provides a basis and impetus towards establishing healthy body fat ranges in Singapore.

### Strength and limitation

The strengths of this study are its population-based, random selection of participants and hence representativeness and validity of data. The ideal method to determine body composition is the multi-compartment model [30], however such method is inaccessible, expensive and require participants to undergo multiple test. DXA, though may have its limitations, has been used in multiple national population surveys and considered the “gold standard” for measuring body composition parameters [31, 46]. There are some limitations to this study. While representative proportions of Malays and Indians were recruited via random sampling, their sample sizes of were too small for ethnic comparisons. Hence, oversampling of Malay and Indian ethnic groups would be needed for ethnic comparison and establishment of a BF% prediction tool. A thorough investigation into the nutrition intake and physical activity may help understand the large discrepancy between BMI and BF%. Future research should utilize a long-term prospective study to define the threshold for obesity, based on the relationship between BF%, all-cause and cause-specific mortality.

In conclusion, our study found a large discrepancy between BF% and BMI measurement in Singaporean adults. The results confirmed that Singaporean adults have higher BF% at lower BMI compared to Caucasians and that BF% in our population have also increased over two decades. Further investigation into the body build, nutrition intake, physical activity level among the different ethnic groups may help understand the relationship between BF% and BMI.

## Supplementary Information

**Additional file 1 **: **Supplementary Table**. Comparing estimated BF% at various BMI point of Singaporean Chinese in this study versus the 1998 National Health Survey.

## Data Availability

The data that support the findings of this study are available from the corresponding author SLW, upon reasonable request. The data are not publicly available due to institutional regulations regarding data containing information that could compromise the privacy of research participants.

## Abbreviations

BMI: Body mass index
BF%: Percentage body fat
DXA: Dual energy x-ray absorptiometry
MOH: Ministry of Health
T2DM: Type 2 Diabetes
US: United States of America
UK: United Kingdom
WC: Waist Circumference
