# Supplementary material for: Relationship between BMI with percentage body fat and obesity in Singaporean adults – The Yishun Study
Source: BMC Public Health. 2021 Jun 1;21:1030. doi: 10.1186/s12889-021-11070-7 (PMC8170923; doi:10.1186/s12889-021-11070-7)
Supplement: Supplementary file 1 — Additional file 1 : Supplementary Table. Comparing estimated BF% at various BMI point of Singaporean Chinese in this study versus the 1998 National Health Survey. [file 12889_2021_11070_MOESM1_ESM.pdf]

Supplementary Table. Comparing estimated BF% at various BMI point of Singaporean Chinese in this study versus the 1998 National Health Survey [1]

	Yishun Study					1998 National Health Survey					Difference in BF%				
	**BMI 18.5**	**BMI 25**	**BMI 30**	**BMI 35**	**BMI 40**	**BMI 18.5**	**BMI 25**	**BMI 30**	**BMI 35**	**BMI 40**	**BMI 18.5**	**BMI 25**	**BMI 30**	**BMI 35**	**BMI 40**
**Men**															
**20-39y**	19.0	27.7	31.8	34.8	37.0	17.0	23.8	29.0	34.2	39.4	2.0	3.9	2.8	0.6	-2.4
**40-59y**	20.8	29.5	33.6	36.6	38.8	19.0	25.8	31.0	36.2	41.4	1.8	3.7	2.6	0.4	-2.6
**60-79y**	22.7	31.3	35.3	38.2	40.4	21.0	27.8	33.0	38.2	43.4	1.7	3.5	2.3	0.0	-3.0
**80+ y**	23.9	32.6	36.7	39.7	41.9	22.5	29.3	34.5	39.7	44.9	1.4	3.3	2.2	0.0	-3.0
**Women**															
**20-39y**	31.8	39.0	42.5	44.9	46.8	27.9	34.7	39.9	45.1	50.3	3.9	4.3	2.6	-0.2	-3.5
**40-59y**	32.8	40.1	43.5	46.0	47.8	29.9	36.7	41.9	47.1	52.3	2.9	3.4	1.6	-1.1	-4.5
**60-79y**	33.8	41.1	44.5	47.0	48.8	31.9	38.7	43.9	49.1	54.3	1.9	2.4	0.6	-2.1	-5.5
**80+ y**	34.6	41.8	45.3	47.7	49.6	33.4	40.2	45.4	50.6	55.8	1.2	1.6	-0.1	-2.9	-6.2

Values presented in percent (%). Difference in BF% is calculated by subtracting values of the 1998 National Health Survey from the current study.

Reference:

1. Deurenberg-Yap M, Schmidt G, Van Staveren WA, Deurenberg P. The paradox of low body mass index and high body fat percentage among Chinese, Malays and Indians in Singapore. International Journal of Obesity 2000; **24**(8): 1011-7
